# Temsirolimus Enhances Anti-Cancer Immunity by Inducing Autophagy-Mediated Degradation of the Secretion of Small Extracellular Vesicle PD-L1

**DOI:** 10.3390/cancers14174081

**Published:** 2022-08-23

**Authors:** Seong-Sik Park, Jong-In Kim, Chan-Hyeong Lee, Ju-Hyun Bae, Ju-Mi Park, Eun-Ji Choe, Moon-Chang Baek

**Affiliations:** Department of Molecular Medicine, CMRI, Exosome Convergence Research Center (ECRC), School of Medicine, Kyungpook National University, Daegu 41944, Korea

**Keywords:** small extracellular vesicle, PD-L1, immunotherapy, temsirolimus, autophagy

## Abstract

**Simple Summary:**

Immune checkpoint blockade therapies (ICBT) have increasing importance in patient survival and prognosis because it enhances immune cell activation by inhibiting the binding of programmed death-ligand 1 (PD-L1) of tumor and programmed death-1 (PD-1) of T cells. However, tumor-derived small extracellular vesicle (sEV) PD-L1 trigger low reactivity in immunotherapy because it promotes tumor growth and metastasis and inhibits activation of immune cell. In this study, temsirolimus (TEM) which the Food and Drug Administration (FDA) approved as a targeted anti-cancer drug, inhibited tumor-derived sEV PD-L1 secretion by activating autophagy. In addition, TEM induced systemic anti-cancer immunity by increasing the number and activation of CD4^+^ and CD8^+^ T cells. Therefore, TEM showed that the anti-cancer effect was better in the breast cancer-bearing-immunocompetent mice than in the nude mice. In summary, we suggest that TEM can overcome sEV PD-L1-mediated immunosuppression in patients with cancer through activation of the immune system in the body by inhibiting tumor-derived sEV PD-L1.

**Abstract:**

Tumor-derived small extracellular vesicle (sEV) programmed death-ligand 1 (PD-L1) contributes to the low reactivity of cells to immune checkpoint blockade therapy (ICBT), because sEV PD-L1 binds to programmed death 1 (PD-1) in immune cells. However, there are no commercially available anti-cancer drugs that activate immune cells by inhibiting tumor-derived sEV PD-L1 secretion and cellular PD-L1. Here, we aimed to investigate if temsirolimus (TEM) inhibits both sEV PD-L1 and cellular PD-L1 levels in MDA-MB-231 cells. In cancer cell autophagy activated by TEM, multivesicular bodies (MVBs) associated with the secretion of sEV are degraded through colocalization with autophagosomes or lysosomes. TEM promotes CD8^+^ T cell-mediated anti-cancer immunity in co-cultures of CD8^+^ T cells and tumor cells. Furthermore, the combination therapy of TEM and anti-PD-L1 antibodies enhanced anti-cancer immunity by increasing both the number and activity of CD4^+^ and CD8^+^ T cells in the tumor and draining lymph nodes (DLNs) of breast cancer-bearing immunocompetent mice. In contrast, the anti-cancer effect of the combination therapy with TEM and anti-PD-L1 antibodies was reversed by the injection of exogenous sEV PD-L1. These findings suggest that TEM, previously known as a targeted anti-cancer drug, can overcome the low reactivity of ICBT by inhibiting sEV PD-L1 and cellular PD-L1 levels.

## 1. Introduction

Programmed death-ligand 1 (PD-L1), an immune checkpoint protein expressed on the surface of cancer cells, induces apoptosis and functional exhaustion of T cells by binding to programmed death-1 (PD-1) protein expressed on the surface of T cells and contributes to cancer growth and metastasis through immune evasion [1,2,3,4]. Therefore, immune checkpoint blockade therapy (ICBT) using monoclonal antibodies such as anti-PD-L1 and anti-PD-1 is effective in cancer treatment through the continuous activation of the body’s immune system and has recently been actively studied [5,6]. However, the response of each patient to ICBT is varied owing to factors such as various cancer types, tumor microenvironment (TME), and tumor-derived small extracellular vesicles (sEVs) [7,8,9]. 

Tumor-derived sEVs are 40–160 nm in size, contain biological molecules (e.g., DNA, RNA, and proteins) of the tumor, and circulate throughout the body through the blood [10,11]. Because of these properties, it has been reported that tumor-derived sEVs can establish a pre-metastatic niche to regulate the TME and facilitate metastasis [12]. Moreover, it was reported that PD-L1 secreted from the tumor sEV binds to PD-1 on immune cells and induces immune evasion, resulting in a low response to ICBT [13,14,15]. Recently, we reported a new paradigm for cancer treatment by identifying mechanisms by which endothelin A (ETA) antagonists improve anti-cancer immunity in the body by inhibiting the secretion of tumor-derived sEV and sEV PD-L1 [16,17,18]. Therefore, the inhibition of sEV secretion and PD-L1 from tumor cells can enhance the response to ICBT. 

A recent study has reported that rapamycin, a drug that inhibits phosphorylation of mammalian target of rapamycin (mTOR), can inhibit the secretion of sEV by activating autophagy within neural cells [19]. Autophagy is a self-digestion process that removes damaged organelles and proteins during various biosynthesis processes and can be activated by inhibiting the phosphorylation of mTOR [20]. Indeed, Xu et al. reported that multivesicular bodies (MVBs), a component of sEV biogenesis, may be degraded by fusion with autophagosomes or lysosomes during autophagy [21]. In addition, rapamycin can inhibit PD-L1 expression in cancer cells, such as gastric cancer and lung cancer, by inhibiting mTOR and activating autophagy [22,23]. Furthermore, Moore et al. reported that the combined administration of rapamycin and anti-PD-L1 to oral cancer-bearing mice effectively confirmed the anti-cancer effect by increasing the activity of CD8^+^ T cells [24]. However, it is not clear whether this phenomenon is the result of inhibition of sEV PD-L1 through autophagy activation. Therefore, we hypothesized that analogs of rapamycin that activate autophagy could induce CD8^+^ T cell-mediated anti-cancer effects by inhibiting the secretion of sEV and sEV PD-L1. 

In this study, we discovered that temsirolimus (TEM), an FDA-approved anti-cancer drug, inhibits the biogenesis and secretion of sEV and sEV PD-L1 by activating autophagy in breast cancer cells, such as MDA-MB-231 cells. In addition, TEM enhanced the activity and counts of CD8^+^ T cells by inhibiting sEV secretion and sEV PD-L1, and cellular PD-L1 expression. Our results confirmed that the combined administration of TEM and anti-PD-L1 antibodies enhanced anti-cancer immunity in a breast cancer model.

## 2. Materials and Methods

### 2.1. Chemicals

FDA-approved mTOR inhibitors, including temsirolimus (HY-50910), rapamycin (HY-10219), and everolimus (HY-10218), were purchased from MedChemExpress (Monmouth Junction, NJ, USA) and used for in vitro and in vivo studies. 

### 2.2. Cell Lines and Cell Culture

All breast cancer and other cells (American Type Culture Collection) were grown at 37 °C in a humidified atmosphere with 5% CO_2_ and 95% air. MCF10A cells were cultured as previously described [16]. MCF7, MDA-MB-231, and A375 cells were cultured in Dulbecco’s Modified Eagle’s Medium with 10% sEV-depleted fetal bovine serum (FBS) and 1% antibiotic-antimycotic solution. SK-MEL-28 cells were cultured in Minimum Essential Medium with 10% sEV-depleted FBS and 1% antibiotic-antimycotic solution. 4T1 cells were cultured in RPMI 1640 supplemented with 10% sEV-depleted FBS and 1% antibiotic-antimycotic solution. FBS was depleted from sEV obtained by ultracentrifugation [25,26]. To analyze the inhibition of sEV secretion by drug treatment, the cells were washed and incubated in a medium containing sEV-depleted FBS. Murine CD8^+^ T cells derived from the spleens of healthy mice were cultured in RPMI 1640 with 20% FBS and 1% antibiotic-antimycotic solution.

### 2.3. Cell Viability Assay

The MDA-MB-231 cells were seeded at approximately 20,000 cells/well in 24-well cell culture plates and incubated for 24 h. The medium was changed to a medium supplemented with exosome-depleted FBS with varying concentrations of mTOR inhibitors, and the cells were grown for 48 h. Then, 0.5 mg/mL MTT [3-(4,5-dimethylthiazole-2-yl)-2, 5-diphenyltetrazolium bromide] solution was added and incubated for 2 h at 37 °C in a humidified atmosphere with 5% CO_2_. After incubation, the MTT solution and medium were aspirated. Dimethyl sulfoxide (DMSO) was then added, and formazan crystals were extracted with gentle shaking at 18–24 °C. The mixtures were transferred to 96-well plates and absorbance was measured at 595 nm using a microplate reader (Multiskan^TM^, ThermoFisher, Waltham, MA, USA).

### 2.4. Isolation of sEVs

sEVs were isolated following the method used in our previous studies [16]. Culture media were collected from cells treated with TEM or vehicle (DMSO). To isolate the sEVs, the media were sequentially centrifuged at 300× *g* for 5 min, 2500× *g* for 15 min, and 10,000× *g* for 30 min. Supernatants were then filtered using 0.2 μm syringe filters. Finally, the supernatants were centrifuged at 120,000× *g* for 90 min using an Optima XE-90 Ultracentrifuge with an SW-28 rotor (Beckman Coulter, Brea, CA, USA). sEV pellets were resuspended in 1 mL phosphate-buffered saline (PBS) or 1 × radioimmunoprecipitation (RIPA) buffer (#50-188, Merch Millipore, Burlington, MA, USA) for further analysis. 

Mouse plasma samples were sequentially centrifuged at 2500× *g* for 15 min and 10,000× *g* for 30 min. The supernatants were subsequently centrifuged at 160,000× *g* for 90 min using an ultracentrifuge with an SW-55Ti rotor (Beckman Coulter, Brea, CA, USA). sEV pellets were homogenized in 1 × RIPA buffer for the analysis of circulating sEV PD-L1.

### 2.5. Transmission Electron Microscopy Analysis

The pure sEVs, isolated by ultracentrifugation, were deposited on pure carbon-coated electron microscopy grids. Next, sEVs were fixed with 2% paraformaldehyde (PFA) for 5 min and washed three times with PBS. For immunogold label staining, sEVs were incubated with mouse anti-PD-L1 antibody (14-5983-82; eBioscience, San Diego, CA, USA). Then, the sEVs were incubated with anti-mouse IgG containing 5 nm gold particles (G7527, Sigma, St. Louis, MO, USA). Finally, the grids were dried at room temperature and stained with 2% uranyl acetate. Visualization was performed at 40,000× magnification using a Hitachi HT-7700 transmission electron microscope (Hitachi, Tokyo, Japan) operating at 100 kV.

### 2.6. Nanoparticle Tracking Analysis (NTA)

The sEV pellets resuspended in PBS were analyzed using a NanoSight LM10 device (NanoSight, Salisbury, UK). To examine the movement and morphology of the nanoparticles, a monochromatic laser beam was set to 405 nm, and a 30 s video was taken at a speed of 30 frames/s and a camera level of 9. During photography preparation of each sample, residual particles were removed by washing the chamber once with 10 mL of PBS to prevent cross-contamination. The size distribution and concentration of the particles were analyzed using NTA software version 2.2 (Nanosight, Salisbury, UK).

### 2.7. Western Blotting

The concentration of cellular or sEV proteins was determined using a bicinchoninic acid (BCA) assay kit (#23227, Thermo Scientific, Waltham, MA, USA) or micro BCA assay kit (#23235, Thermo Scientific). The proteins were resolved by sodium dodecyl sulfate-polyacrylamide gel electrophoresis (SDS-PAGE), transferred onto nitrocellulose membranes, bound with primary antibodies, and incubated with horseradish peroxidase (HRP)-linked secondary antibody. Images were visualized using enhanced chemiluminescence (ECL) detection reagents (#34580, Thermo Scientific) with ECL hyper film (AGFA, Morstel, Belgium) and a Fusion FX7 system (Vilber Lourmat, Eberhardzell, Germany). Western blots were normalized to β-actin in the whole cell lysate and CD63 in the proteins of sEV. Densitometric analysis was performed using image J. The following primary antibodies were used: PD-L1 (#13684, CST), Flotilin-1 (#3253, CST), TSG101 (ab30871, Abcam, Cambridge, UK), CD63 (ab68418, Abcam), Alix (#2171, CST), Syntenin-1 (ab133267, Abcam), Rab11 (ab18211, Abcam), Rab7 (ab50533, Abcam), Rab27A (ab55667, Abcam), Rab27B (ab76779, Abcam), LC3 (NB600-1384, Novus, Stroudsburg, PA, USA), p62 (#5114, CST), Beclin-1 (#3738, CST), and β-actin (SC-47778, Santa Cruz Biotechnology, Dallas, TX, USA). All the whole western blot figures can be found in the Supplementary Materials (Figure S1).

### 2.8. Immunofluorescence Staining

MDA-MB-231 cells were seeded onto glass coverslips at approximately 1 × 10^4^ cells/well in a six-well confocal chamber overnight and treated with TEM for 24 h. To observe lysosomal activity, 75 nM Lysotracker (L7528, Thermo Scientific) was added to the culture medium for 1 h before fixing with 4% PFA in PBS for 15 min at room temperature. Then, they were washed three times with PBS for 5 min, and the coverslips were stained and mounted using Prolong Gold Antifade Mountant with 4′,6-diamidino-2-phenylindole (DAPI) (P36931, Invitrogen, Waltham, MA, USA). To investigate the fusion of autophagosomes or lysosomes with MVBs, LC3 (NB600-1384, Novus), LAMP-1 (ab25630, Abcam), and CD63 (ab8219, Abcam) were used as autophagosomes, lysosomes, and MVBs markers, respectively. Colocalization of LC3 and LAMP-1, LC3 and CD63, and LAMP-1 and CD63 were quantified as Pearson’s correlation coefficients using ImageJ [27].

### 2.9. CD8^+^ T Cell-Mediated Cancer Cell Killing Assay

Murine CD8^+^ T cells were isolated from the spleen of healthy BALB/c mice. First, CD8^+^ T cells were activated by incubation with 2 µg/mL mouse CD3/CD28 antibody and 150 IU/mL IL-2 for 24 h. Next, 4T1-luciferase cells were seeded in 96-well plates at a density of approximately 1000 cells/well. After 12 h, the 4T1-luciferase cells were co-cultured with murine CD8^+^ T cells at an effector-to-target (E:T) ratio of 1:5 and then treated with or without drugs and anti-PD-L1 for 48 h. The plates were washed with PBS and 100 µL of 2 mg/mL luciferin was added to each well. Luciferase intensity in each well was immediately measured using an Alpha microplate reader (PerkinElmer, Waltham, MA, USA). 

### 2.10. Enzyme-Linked Immunosorbent Assay (ELISA)

After the CD8^+^ T cell-mediated cytotoxicity assay, the supernatants of each well were centrifuged at 300× *g* for 5 min and the cell debris was removed and used for ELISA. To measure cytokine levels, anti-mouse TNF-α ELISA kits (#MTA00B; R&D Systems, Minneapolis, MN, USA) were used. All processes were performed according to the manufacturer’s instructions. 

### 2.11. Flow Cytometric Analysis for Immune Phenotyping

At the end of the animal experiments, tumors were harvested, mechanically cut into small pieces, and digested into a single-cell suspension using a mouse tumor dissociation kit (Miltenyi Biotec, Bergisch Gladbach, Germany) according to the manufacturer’s instructions. Dead cells isolated from tumors and draining lymph nodes (DLNs) were stained using the Fixable Aqua Dead Cell Stain Kit (Invitrogen). Cell surface/intracellular markers were stained, and immune phenotypes in the tumors and DLNs were analyzed as previously described [19]. The following fluorophore-conjugated antibodies were used in this analysis: efluor780 anti-mouse CD45, peridinin chlorophyll protein-cyanine 5.5 anti-mouse CD4, efluor450 anti-mouse CD8, allophycocyanin (APC) anti-mouse Tim3 (eBioscience), fluorescein isothiocyanate (FITC) anti-mouse CD3, phycoerythrin (PE) anti-mouse FoxP3, and APC anti-mouse IFNγ (BioLegend).

### 2.12. Animal Studies

All animal experiments were performed according to protocols approved by the Kyungpook National University (KNU) Institutional Animal Care and Use Committees (IACUCs; Approve number: 2022-141). Five- to six-week-old nude (BALB/cAnNCrl-nuBR) and BALB/c (BALC/cAnNcrl) mice were purchased from Orient Bio (Seongnam, Korea). The mice were housed in a pathogen-free facility. For the analysis of circulating sEVs after drug treatment, approximately 2 × 10^4^ 4T1 cells suspended in 100 µL of PBS containing 50% Matrigel (Corning) were orthotopically injected into the left fat pad of the mice. TEM was intraperitoneally injected once every 3 days. Anti-mouse PD-L1 (Bio X Cell, Lebanon, NH, USA) was intraperitoneally injected at 200 μg/mouse once every 3 days three times. In rescue experiments, 10 µg of 4T1-derived sEVs was intravenously injected into the tail vein once every 3 days three times. The sEV-associated PD-L1 was blocked by using the same antibody administered to the animal [13]. During animal experiments, tumor volume was recorded until the maximum value defined in the IACUC guidelines was reached. When the tumor reached an average size of 50–100 mm^3^, the mice were randomized into groups with similar distributions of starting tumor volume. The mice were euthanized when the tumor volume reached 1500 mm^3^. The tumor volume was estimated using calipers and calculated for each mouse using the following equation: volume (cm^3^) = width^2^ × length × 0.5.

To investigate the PD-L1 level of tumor cells, tumor samples were harvested and mechanically cut into pieces less than 2 mm in length. Subsequently, 1 × RIPA buffer was added, reacted at 4 °C for 30 min, and then centrifuged for 30 min to harvest supernatant.

### 2.13. Statistical Analysis

Statistical analyses were performed using GraphPad Prism version 6.0. Statistical significance of the experimental results was estimated using an unpaired two-tailed Student’s *t*-test. Error bars in the graph represent the mean ± standard deviation. All in vitro experiments were performed in triplicate unless otherwise stated. Tumor volume and immune phenotyping data are represented as the mean ± standard error of the mean (SEM). Statistical significance was set at *p* < 0.05. *, **, and *** denote *p*-values of <0.05, 0.01, and 0.001, respectively.

## 3. Results

### 3.1. TEM Inhibits sEV PD-L1 Secretion through the Additive Effect of Suppressing sEV Secretion and Cellular PD-L1 Expression

**Figure 1:**
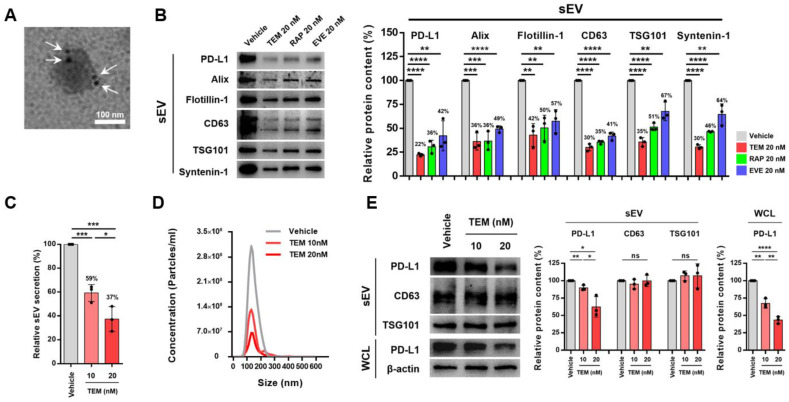
Temsirolimus (TEM) suppresses sEV PD-L1 through the inhibition of small extracellular vesicle (sEV) secretion and cellular PD-L1 level. (**A**) A transmission electron microscopy image of MDA-MB-231-derived sEVs conjugated with immunogold-labeled PD-L1 antibodies. Arrowheads indicate 5 nm gold particles. Scale bar, 100 nm. (**B**) The immunoblots of various proteins in sEV from MDA-MB-231 with or without treatment of mTOR inhibitors (**left**) (*n* = 3). sEV proteins from approximately 1.2 × 10^7^ cells were loaded per lane. The quantitative analysis of the relative protein expression (**right**). (**C**) The relative percentage of secreted sEV from MDA-MB-231 cells with or without treatment of TEM by nanoparticle tracking analysis (NTA) (*n* = 3). (**D**) A representative graph of the size distribution of secreted sEV from MDA-MB-231 cells with or without treatment of TEM. (**E**) Immunoblots of various proteins in sEV and whole cell lysate (WCL) from MDA-MB-231 cells with or without treatment of TEM (**left**) (*n* = 3). An equal protein weight (3 μg) of the sEV was loaded per lane. β-actin was used as the loading control. The quantitative analysis of the relative protein expression (**right**). The data are presented as means ± standard deviation (SD) using an unpaired two-tailed Student’s *t*-test. * *p* < 0.05, ** *p* < 0.01, *** *p* < 0.001, and **** *p* < 0.0001, respectively; ns, not significant.

Tumor-derived sEV PD-L1 binds to PD-1 on CD8^+^ T cells, resulting in a low response to ICBT. Thus, we hypothesized that the activation of autophagy through the inhibition of mTOR signaling enhances the response to ICBT by suppressing the level of sEV PD-L1. First, we analyzed the cell line with the highest sEV PD-L1 secretion to confirm the inhibition of sEV PD-L1 secretion by mTOR inhibitors (TEM, rapamycin, and everolimus). As a result, we determined that the sEV PD-L1 level, as well as the cellular PD-L1 level, was the highest in MDA-MB-231 cells, a breast cancer cell line, and selected this as the cell line to be used in this study (Figure S2A). Furthermore, we confirmed the morphology of sEV and sEV PD-L1 secreted from MDA-MB-231 cells (Figure 1A). Next, to confirm the inhibition of sEV PD-L1 secretion by treatment with an mTOR inhibitor, a concentration of 20 nM or less was used to exclude apoptotic bodies, to this concentration does not affect cell viability (Figure S2B). Treatment with mTOR inhibitors suppressed the levels of sEV PD-L1 derived from the same number of MDA-MB-231 cells and reduced the levels of sEV marker proteins, such as Alix, Flotillin-1, CD63, TSG101, Syntenin-1 (Figure 1B). In particular, TEM effectively inhibited the level of sEV PD-L1 in a dose-dependent manner compared with other mTOR inhibitors. Interestingly, the inhibition of the levels of sEV PD-L1 by TEM treatment [approximately 46% (10 nM), 80% (20 nM)] was higher than the inhibition of the levels of sEV marker proteins such as CD63 and TSG101 [approximately 34% (10 nM), 61% (20 nM)]. Similar to other mTOR inhibitors, TEM most strongly inhibited sEV secretion at 41% (10 nM) and 63% (20 nM) in a dose-dependent manner without changing the size distribution (Figure 1C,D and Figure S2C–E). Consistent with these results, the levels of sEV marker proteins were inhibited by TEM in 4T1 murine breast cancer cells and A375 human melanoma cells (Figure S3A,B). These results are similar to those of inhibition of sEV marker proteins by TEM in the Western blot (Figure 1B). A previous study has reported that mTOR inhibition by rapamycin treatment inhibits cellular PD-L1 levels in non-small cell lung cancer cells [22]. TEM suppressed not only the cellular PD-L1 levels in MDA-MB-231 cells, 4T1 cells, and A375 cells but also reduced the same amount of PD-L1 levels of sEVs in a dose-dependent manner (Figure 1E and Figure S3C). Taken together, these results suggest that TEM suppresses sEV PD-L1 levels through the additive effect of inhibiting sEV secretion and cellular PD-L1 levels (Figure 1B).

### 3.2. TEM Suppresses sEV PD-L1 Secretion through the Regulation of Rab Protein Levels and Activation of Autophagy

**Figure 2:**
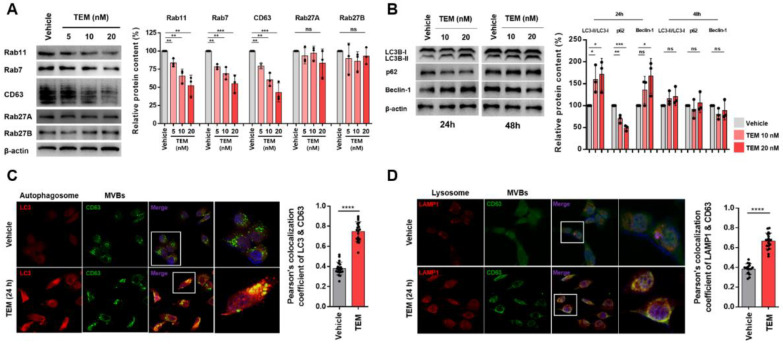
Temsirolimus (TEM) induces degradation of multivesicular bodies (MVBs) through regulation of Rab proteins and activation of autophagy. (**A**,**B**) The immunoblots of various proteins are associated with (**A**) small extracellular vesicle (sEV) secretion and (**B**) autophagy in MDA-MB-231 cells. (**left**) (*n* = 3). β-actin was used as the loading control. The quantitative analysis of the relative protein expression (**right**). (**C**) Confocal microscopy image. Colocalization of LC3 (autophagosome) and CD63 [(multivesicular bodies (MVBs)] in MDA-MB-231 cells by TEM treatment. (**D**) Confocal microscopy image. Colocalization of LAMP1 (lysosome) and CD63 (MVBs) in MDA-MB-231 by TEM treatment. The colocalization was estimated using Pearson’s correlation coefficient. The data are presented as means ± standard deviation (SD) using an unpaired two-tailed Student’s *t*-test. * *p* < 0.05, ** *p* < 0.01, *** *p* < 0.001, and **** *p* < 0.0001, respectively; ns, not significant.

We investigated whether TEM inhibits sEV PD-L1 secretion by altering the expression of proteins involved. sEVs are generated intracellularly as intraluminal vesicles within MVBs, which are released upon docking/fusion of the MVB with the plasma membrane [11]. In the whole-cell lysate (WCL) of cells (MDA-MB-231 and 4T1) treated with TEM for 48 h, the levels of Rab7 and CD63, which are MVB marker proteins, and Rab11, a protein involved in the docking of the MVBs to the plasma membrane, were all decreased. However, the levels of Rab27A and Rab27B, the main regulators of sEV secretion, showed no significant change (Figure 2A and Figure S4A). These results suggest that TEM not only caused a decrease in the number of MVBs in the cells, but also that the secretion of sEV was inhibited by preventing the docking of MVBs to the cell membrane. Next, we examined whether TEM suppressed the secretion of sEV by inducing autophagy activation. A correlation between autophagy and sEV secretion has been reported: sEV secretion may be inhibited by MVB fusion with autophagosomes and lysosomes involved in autophagy [21]. First, we treated MDA-MB-231 cells with TEM for 24 and 48 h at each concentration, and then examined the changes in the levels of proteins such as microtubule-associated protein light chain 3 (LC3), beclin-1, and SQSTM1 (p62), which are closely related to autophagy activation. Once autophagy is activated, LC3 is conjugated and converted from LC3-I (16 kDa) to LC3-II (14 kDa), thus making the amount of LC3-II a significant indicator of autophagosome formation [28]. p62 is one of the substrates of autophagy and is degraded during autophagy [29]. Beclin 1 acts as an essential mediator of autophagy [30]. As a result, we confirmed that when the cells were exposed to TEM for 24 h, autophagy was activated (Figure 2B and Figure S4B). On the contrary, when the cells were treated with TEM for 48 h, autophagy activation was not affected (Figure 2B). Moreover, the activity of autophagy and lysosomes was increased when cells were exposed to TEM for 24 h (Figure S4C,D). Next, we confirmed by performing immunocytochemistry that MVBs were degraded by colocalization between autophagosomes (LC3B) and MVBs or lysosomes (LAMP1) and MVBs following TEM treatment of MDA-MB-231 for 24 h (Figure 2C,D). Similar to these results, the MVBs of 4T1 cells were degraded by colocalization between autophagosomes and MVBs when the cells were exposed to TEM for 24 h (Figure S4E). On the other hand, the degradation of MVBs through colocalization between lysosome and MVBs in 4T1 cells exposed to TEM for 24 h was not significantly observed (Figure S4F). These results suggest that the secretion of sEV and sEV PD-L1 is suppressed by inducing autophagy-mediated MVB degradation by TEM treatment.

### 3.3. TEM Improves CD8^+^ T Cell-Mediated Anti-Cancer Effects

**Figure 3:**
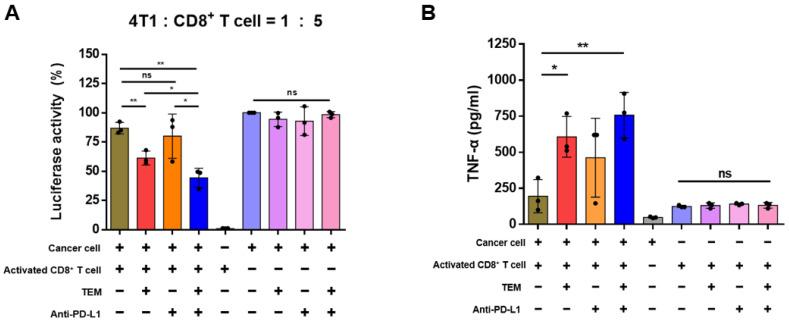
Temsirolimus (TEM) promotes CD8^+^ T cell-mediated anti-cancer immunity. (**A**) The murine CD8^+^ T cell-mediated cancer killing effect in the 4T1-luciferase cells after indicated treatments (*n* = 3). (**B**) Cytokine levels of TNF-α in the supernatant after co-culture with CD8^+^ T cells and cancer cells or in the supernatant of each cell alone were quantified using enzyme-linked immunosorbent assay (ELISA) (*n* = 3). The data are presented as means ± standard deviation (SD) using an unpaired two-tailed Student’s *t*-test. * *p* < 0.05, ** *p* < 0.01, respectively; ns, not significant.

sEV PD-L1 can induce immunosuppression by binding to PD-1 on the surface of CD8^+^ T cells through body circulation [13,19]. Thus, we performed a CD8^+^ T cell-mediated cancer cell killing assay to confirm that TEM treatment can increase the CD8^+^ T cell-mediated anti-cancer effect by inhibiting the secretion of sEV PD-L1. In the co-culture of activated CD8^+^ T cells and 4T1 cancer cells, TEM alone or in combination with anti-PD-L1 improved CD8^+^ T cell-mediated cancer cell killing. On the contrary, TEM alone or in combination with anti-PD-L1 antibodies did not exhibit a direct cytotoxic effect on 4T1 cells (Figure 3A). These results suggest that TEM increased the activity of CD8^+^ T cells by inhibiting sEV PD-L1 secretion, and anti-cancer effects were exhibited owing to an additive effect with anti-PD-L1. We also performed a cytokine ELISA to confirm whether TEM alone or in combination with anti-PD-L1 induced the activation of CD8^+^ T cells. Tumor growth may be inhibited by the pro-inflammatory milieu generated by effector cytokines such as TNF-α, which are released by CD8^+^ T cells [31]. In the co-culture of activated CD8^+^ T cells and 4T1 cancer cells, TEM alone or in combination with anti-PD-L1 effectively increased the release of TNF-α from CD8^+^ T cells (Figure 3B). These results suggest that TEM can induce CD8^+^ T cell-mediated anti-cancer effects by inhibiting the secretion of sEV PD-L1 in cancer cells and promoting the release of cytokines, such as TNF-α, in CD8^+^ T cells.

### 3.4. TEM Enhances the Efficacy of Anti-PD-L1 Therapy

**Figure 4:**
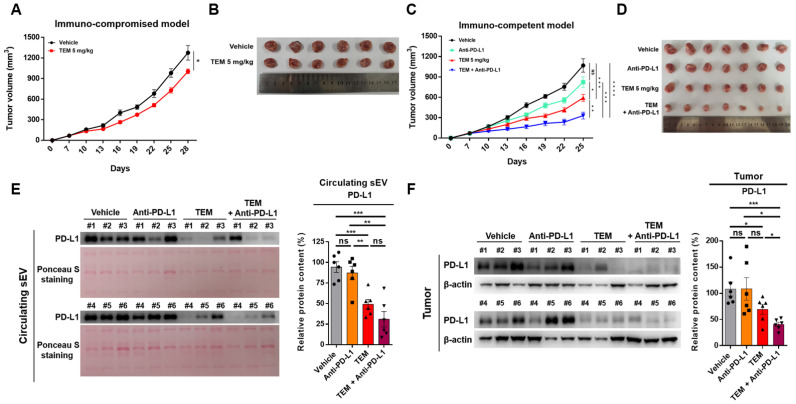
Temsirolimus (TEM) augments anti-cancer effects by inhibiting sEV PD-L1 and cellular PD-L1 levels in the 4T1 tumor model. (**A**–**D**) The growth curves (**left**) and tumor image (**right**) of 4T1 breast cancer in (**A**,**B**) immunocompetent mice and (**C**,**D**) nude mice (*n* = 6–7). (**E**) The immunoblots of PD-L1 of the circulating sEV and (**F**) cellular PD-L1 levels in tumors treated with or without TEM (**left**). The quantitative analysis of the relative protein expression (**right**). The data are presented as means ± standard error of the mean (SEM). * *p* < 0.05, ** *p* < 0.01, *** *p* < 0.001, and **** *p* < 0.0001, respectively; ns, not significant.

Next, we used the 4T1 tumor model to investigate whether TEM exhibits anti-cancer effects through the activation of immune cells by reducing the protein levels of circulating sEV PD-L1 and cellular PD-L1. First, we determined the route of administration and dosage of TEM. TEM was developed as an mTOR-targeted anti-cancer drug in a patient with renal cancer and has been administered intraperitoneally in many studies. Moreover, a 5 mg/kg dose of TEM was selected for the animal study because this dose has been used for various types of cancer therapy [32,33]. When TEM was administered at a dose of 5 mg/kg to immunocompromised mice, tumor growth was reduced by approximately 22% compared to that in the vehicle group (Figure 4A,B). These results suggest that TEM suppresses mTOR levels in cancer cells, thereby inhibiting the proliferation of cancer cells and thus exhibiting anti-cancer effects [32,33]. Next, when administered to immunocompetent mice at a dose of 5 mg/kg, tumor growth was inhibited by approximately 45% compared to that in the vehicle group (Figure 4C,D). The reason anti-cancer effects are more effective in immunocompetent mice is that TEM suppresses cancer cell proliferation by inhibiting mTOR levels and promoting immune cell-mediated anti-cancer effects. The inhibition of tumor growth by a single administration of anti-PD-L1 antibodies was not significant compared to that in the vehicle group. However, co-administration of TEM and anti-PD-L1 antibodies strongly inhibited tumor growth by approximately 71% (Figure 4C,D). Next, we examined whether TEM alone or in combination with anti-PD-L1 inhibited sEV PD-L1 secretion and tumor PD-L1 expression. The protein levels of PD-L1 in circulating sEV and tumor lysates isolated from 4T1-bearing immunocompetent mice treated with TEM alone or in combination with anti-PD-L1 were significantly reduced. (Figure 4E,F). These results show that TEM reduced the protein levels of tumor-derived sEV PD-L1 and cellular PD-L1. Therefore, our results suggest that TEM could inhibit the growth of tumors in immunocompetent patients and improves the efficacy of anti-PD-L1 therapy.

### 3.5. Combined Administration of TEM and Anti-PD-L1 Boosts Anti-Cancer Immunity

**Figure 5:**
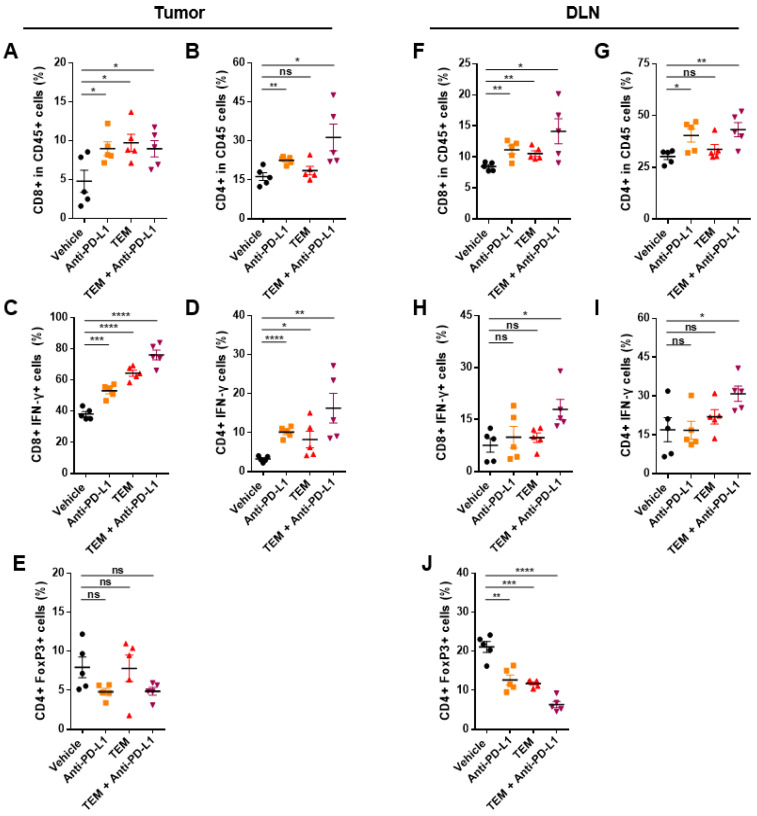
Combined administration of TEM and anti-PD-L1 antibodies increases the immune response in the 4T1 tumor model. (**A**–**J**) Flow cytometric analysis of the T lymphocytes in tumors and draining lymph nodes (DLNs) (*n* = 6). The proportions of (**A**) CD8^+^ cells and (**B**) CD4^+^ cells in the CD45^+^ cells in the tumor. The proportions of IFN-γ producing cells among the (**C**) CD8^+^ cells and (**D**) CD4^+^ cells in the tumors. The proportions of (**E**) CD4^+^ FoxP3^+^ cells in the tumors. The proportions of (**F**) CD8^+^ cells and (**G**) CD4^+^ cells of the CD45^+^ cells in the DLNs. The proportions of IFN-γ cells among the (**H**) CD8^+^ cells and (**I**) CD4^+^ cells in the DLNs. The proportions of (**J**) CD4^+^ FoxP3^+^ cells in the DLNs. The data are presented as means ± standard error of the mean (SEM). * *p* < 0.05, ** *p* < 0.01, *** *p* < 0.001, and **** *p* < 0.0001, respectively; ns, not significant.

Next, we confirmed whether immune cells such as cytotoxic T cells, helper T cells, and regulatory T cells in tumors and DLNs were activated by TEM alone or in combination with anti-PD-L1 in the 4T1-bearing mouse model (Figure S5A,B). The levels of cytotoxic activity factors such as IFN-γ of CD8^+^ T cells and populations of CD4^+^ T cells were significantly increased in the tumors of the mice co-administered TEM and anti-PD-L1 antibodies (Figure 5A–D). On the contrary, the levels of regulatory T cell (Treg) markers, such as Foxp3, which induces immunosuppression, did not show significant changes compared to that in the vehicle group (Figure 5E). In DLNs, as in tumors, the population and activity of CD8^+^ and CD4^+^ T cells were increased by the combined administration of TEM and anti-PD-L1 antibodies. (Figure 5F–I). Interestingly, the population of Tregs in DLNs was reduced by the co-administration of TEM and anti-PD-L1 antibodies (Figure 5J). These results show that the combined administration of TEM with anti-PD-L1 antibodies induces an increase in the number and activity of immune cells and enhances overall systemic anti-cancer immunity.

### 3.6. Combined Administration of TEM and Anti-PD-L1 Antibodies Reverses sEV PD-L1-Mediated Tumor Growth

**Figure 6:**
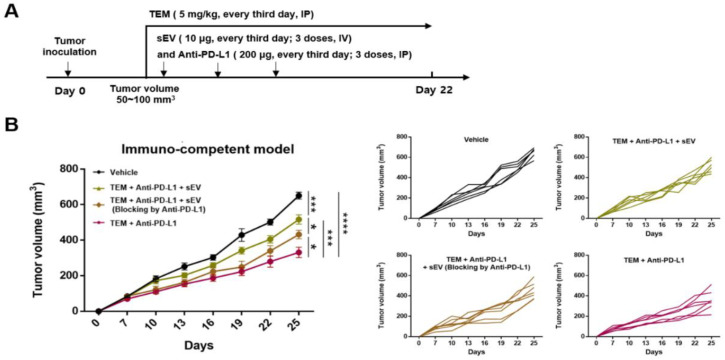
The addition of exogenous sEV PD-L1 reverses the anti-cancer effect induced by TEM and anti-PD-L1 antibodies. (**A**) The design of the tumor rescue experiment with exogenous sEV PD-L1 injections in syngeneic 4T1-bearing models. (**B**) The growth curves of 4T1 tumors in immunocompetent mice injected with the indicated treatment (*n* = 6–7). The data are presented as means ± standard error of the mean (SEM). * *p* < 0.05, *** *p* < 0.001, and **** *p* < 0.0001, respectively; ns, not significant.

In the 4T1-bearing immunocompetent mouse model, the combined administration of TEM and anti-PD-L1 antibodies induced anti-cancer immunity, which activated T cells by inhibiting circulating sEV PD-L1 and cellular PD-L1 levels. Next, we studied whether this anti-cancer effect could be reversed by injecting exogenous sEV PD-L1 (Figure 6A). We intravenously injected 4T1-derived sEVs into 4T1-bearing immune-competent mice cotreated with TEM and anti-PD-L1 antibodies. Both the groups injected with sEVs (TEM + anti-PD-L1 + sEV) and the group injected with anti-PD-L1 (TEM + anti-PD-L1 + sEV blocked by anti-PD-L1) showed tumor growth recovery compared to the group injected with a combination of TEM and PD-L1 antibodies (Figure 6B).

## 4. Discussion

TEM is an FDA-approved targeted anti-cancer drug that limits the survival of cancer cells and promotes autophagy by inhibiting phosphorylation of mTOR in cancer cells [34]. In addition, TEM is a water-soluble rapamycin analog that improves the solubility and low pharmacokinetic properties of rapamycin, which has been previously used as an antifungal and anti-cancer agent [35]. mTOR is an essential regulator of cancer cell response to growth factors, proliferation, and survival. In addition, mTOR activity is upregulated in human cancer and can accelerate tumorigenesis and development through various mechanisms, such as angiogenesis, promotion of growth factor receptor signaling, and suppression of autophagy [34,35]. In fact, TEM significantly improved the overall survival rate and prognosis of patients with renal cancer in clinical trials [34]. In addition, in preclinical trials, TEM has been studied as a drug that can enhance the anti-cancer effect in various cancers, such as pancreatic cancer, neuroectodermal tumor, and medulloblastoma [32,33]. 

Here, we demonstrated a novel mechanism by which TEM not only exhibits tumor-suppressive effects on breast cancer cells but also improves CD8^+^ T cell-mediated anti-cancer effects through the inhibition of breast cancer-derived sEV PD-L1 levels. In addition, the number and activity of CD4^+^ and CD8^+^ T cells were increased in the tumors and DLNs of breast cancer-bearing immunocompetent mice by the combined administration of TEM and anti-PD-L1, thereby inducing anti-cancer immunity. Therefore, we believe that TEM, previously known as a targeted anti-cancer drug, can be developed as a new anti-cancer therapy that can effectively enhance cancer treatment by suppressing sEV PD-L1 secretion to improve the immune system in the body.

Tumor-derived sEV PD-L1 is considered to be a factor that attenuates the response of ICBT in patients with cancer [36,37]. In fact, the release of sEV PD-1 was suppressed in a cancer cell model in which the *RAB27A* and *nSMase2* genes, which are related to sEV secretion, were deleted, thereby increasing the response to ICBT through the activation of immune cells [14]. Nevertheless, among commercially available chemical anti-cancer drugs or small-molecule anti-cancer drugs, no drug exists that inhibits both tumor-derived sEV PD-L1 secretion and cellular PD-L1. According to our findings, TEM, which has already been proven safe and commercialized, could be rapidly applied as a new drug for cancer immunotherapy that activates the immune system by inhibiting sEV PD-L1 secretion. TEM has been shown to have anti-cancer effects that exhibit a favorable toxicity profile and cancer-resistant efficacy in the treatment of patients with gastric cancer in phase I of clinical trials [34]. Here, we activated anti-cancer immunity by inhibiting sEV PD-L1 secretion through an intraperitoneal injection at 5 mg/kg in breast cancer-bearing mouse models. Therefore, it is necessary to assess the anti-cancer effects of TEM to activate the immune system by inhibiting sEV PD-L1 secretion in various cancer models.

As a strategy to overcome the low response to ICBT by inhibiting sEV PD-L1 secretion in patients with cancer, the following steps were considered: (1) suppression of sEV secretion, and/or (2) suppressing cellular PD-L1 levels. Recently, it is reported that inhibiting sEV PD-L1 secretion could improve the efficacy of anti-PD-L1 and anti-cancer immunity because tumor-derived sEV and sEV PD-L1 contribute to tumor growth and immune evasion by inhibiting T cell activity, such as NF-κB, IL-2, and IFN-γ activation [38,39]. Furthermore, we previously confirmed that suppression of sEV PD-L1 secretion through the inhibition of tumor-derived sEV secretion by the ETA antagonists sulfisoxazole and macitentan caused the activation of immune cells, thereby exhibiting anti-cancer effects [16,17,18]. Next, it is known that inhibition of PD-L1 expressed by cancer cells can activate the immune system in the body [39]. For example, tipifarnib is a farnesyltransferase inhibitor targeting HRAS that increases anti-cancer effects through the prevention of apoptosis of T cells and downregulation of the level of cellular PD-L1 of renal cancer cells [40]. We confirmed that TEM inhibited both sEV PD-L1 and cellular PD-L1 levels, strongly suggesting that it has the potential to effectively overcome the low reactivity to ICBT in patients.

Recently, it has been reported that autophagy, negatively regulated by mTOR, is involved in the inhibition of sEV secretion. Autophagy is a self-degradation process in which cytoplasmic components are fused in double-membrane vesicles, such as autophagosomes, and transferred to lysosomes for degradation [19,20,21]. Induction of autophagy has been shown to inhibit the secretion of sEVs by promoting colocalization of MVBs with autophagosomes, whereas impaired autophagy may lead to increased secretion of sEVs [20]. Therefore, inhibition of the secretion of tumor-derived sEVs containing PD-L1 through autophagy activation by TEM could be effective for cancer treatment. In addition, Hao et al. reported that autophagy inhibition by genetic deletion of an essential autophagy gene, such as Fip200, increases the secretion of sEV in her2-positive breast cancer [41]. On the contrary, Zou W et al. reported that inhibition of mTOR, which negatively regulates autophagy, stimulates the secretion of sEV in mouse embryo fibroblasts [42]. These studies show that autophagy functions as a double-edged sword in the secretion of sEV. As mentioned above, the secretion of sEV may differ according to the type of cell, stage of autophagy, and genetic alteration of autophagy. Thus, in future studies, it will be necessary to confirm the change in sEV secretion according to genetic changes in autophagy in various cancer cells.

Regulation of autophagy is involved not only in the secretion of sEV but also in the regulation of cellular PD-L1 levels [23,43]. One study reported that the inhibition of autophagy by pharmacological chemicals or small interfering RNAs (siRNAs) upregulated the levels of PD-L1 in gastric cancer cells and xenografts [23]. Another study found that a new HDAC6 inhibitor, MPT0G612, activated autophagy to downregulate PD-L1 levels in colorectal cancer [43]. Therefore, TEM, an activator of autophagy, may improve the efficacy and prognosis of cancer treatment by downregulating sEV PD-L1 and cellular PD-L1 levels. 

We confirmed that the number and activity of CD8^+^ and CD4^+^ T cells in tumors and DLNs of the 4T1-bearing immunocompetent model were both increased by the combined administration of TEM and anti-PD-L1 antibodies, thereby exhibiting anti-cancer effects. We also found that combined administration of TEM and anti-PD-L1 increased the number and activity of CD8^+^ T cells and this is consistent with a recent report that mTOR inhibition and combination therapy with an immune checkpoint inhibitor can enhance the anti-cancer effect [24,44]. For example, in oral cavity cancer, the production of cytokines such as IFN-γ in peripheral and tumor-infiltrating CD8^+^ T cells and expression of MHC class 1 were increased by combination therapy with rapamycin and anti-PD-L1 [24]. Similarly, in the colon cancer model, the combination of vistusertib, an mTOR inhibitor, and anti-CTLA-4, an immune checkpoint blockade, increased the cytotoxic activity of intratumoral CD8^+^ T cells, by the production of molecules such as IFN-γ and granzyme B [43]. These results may also include the effects of the inhibition of sEV PD-L1 or cellular PD-L1 levels by mTOR inhibition in cancer. 

The number and activity of CD4^+^ T cells in both tumors and DLNs were not effectively increased by TEM alone; however, the combination of TEM and anti-PD-L1 antibodies both effectively increased the number and activity of CD4^+^ T cells. Moreover, we found that the populations of Tregs in DLNs were decreased by the combined administration of TEM and anti-PD-L1. However, no significant change in the number of Tregs was observed in the tumors. Many studies related to mTOR have reported that inhibition of mTOR increases the number of Tregs, which suppresses the immune response [45,46]. However, Chapman et al. reported that inhibition of mTOR by pp242, an mTOR inhibitor, prevented the suppressive activity of Tregs [47]. Indeed, in a patient with renal cancer, it was reported that the number of effector Tregs significantly decreased when changes in immune cells were analyzed after the administration of everolimus, another rapamycin analog, and TEM [48]. In addition, breast cancer-derived sEVs containing transforming growth factor-beta (TGF-β), which mediate the proliferation of Tregs, are involved in immunosuppression [49,50]. Thus, our results showing anti-cancer immunity through the inhibition of sEV PD-L1 by TEM treatment may support the results of previous studies. However, because it is unclear how TEM affects immune cell-derived sEVs and other immune cells, additional mechanistic studies are needed.

## 5. Conclusions

In summary, although tumor-derived sEV and cellular PD-L1 are factors that reduce the reactivity to ICBT in patients with cancer, there are no commercially available anti-cancer drugs that inhibit all of them. We demonstrated that the induction of autophagy by TEM treatment in cancer cells is an important molecular mechanism for suppressing sEV PD-L1 and cellular PD-L1 levels. In addition, TEM enhanced the CD8^+^ T cell-mediated anti-cancer effect in both the co-culture of cancer and CD8^+^ T cells and the breast cancer-bearing mouse model. Moreover, it induces anti-cancer-immunity by enhancing the efficacy of anti-PD-L1 therapy. Therefore, TEM may be developed as a drug that can enhance the response to ICBT by overcoming tumor-derived sEV PD-L1- and cellular PD-L1-mediated immunosuppression.

## Data Availability

All other relevant data of this study are available from the corresponding authors upon reasonable request.

## Supplementary Materials

The following supporting information can be downloaded at: www.mdpi.com/article/10.3390/cancers14174081/s1, Figure S1: Original blots of Western blot analyses in this paper; Figure S2: mTOR inhibitors inhibit secretion of sEV from MDA-MB-231 cells; Figure S3: TEM reduces sEV PD-L1 secretion via the additive effect of inhibiting sEV secretion and cellular PD-L1 expression in the various cancer cell lines; Figure S4: TEM induces activation of autophagy; Figure S5: The gating strategy used in flow cytometry analysis
