# Supplementary material for: Temsirolimus Enhances Anti-Cancer Immunity by Inducing Autophagy-Mediated Degradation of the Secretion of Small Extracellular Vesicle PD-L1
Source: Cancers (Basel). 2022 Aug 23;14(17):4081. doi: 10.3390/cancers14174081 (PMC9454510; doi:10.3390/cancers14174081)
Supplement: Supplementary file 1 [file cancers-14-04081-s001.zip › cancers-1835695-supplementary.pdf]

cancers

MDPI

## Supplementary figures

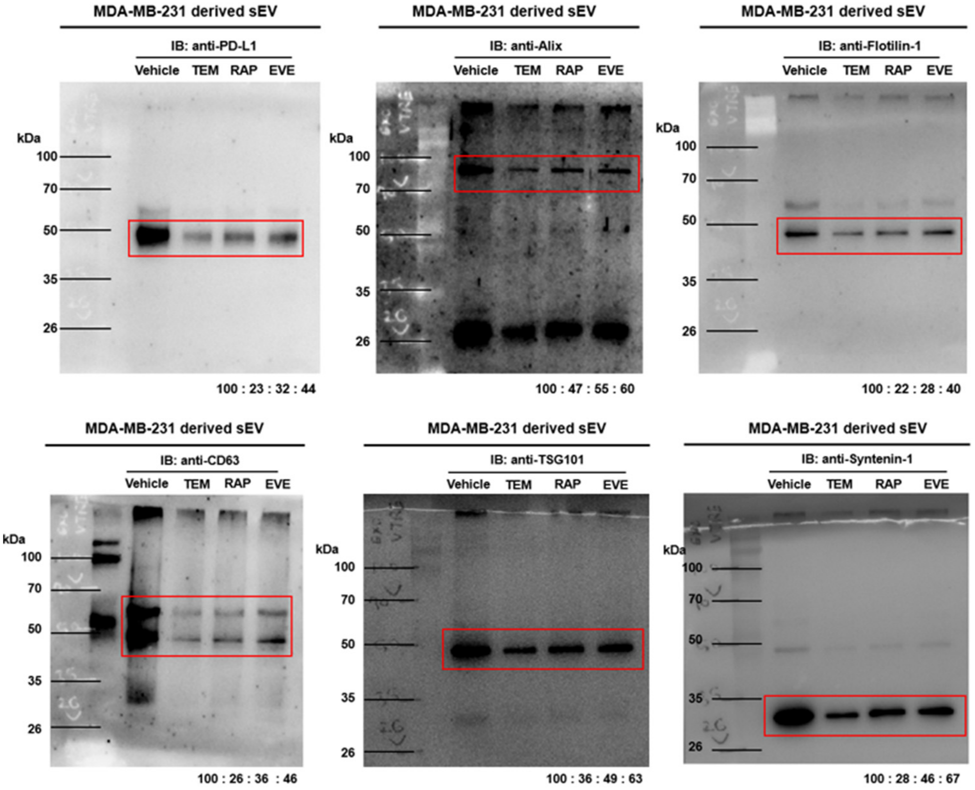


**Figure 1. B**

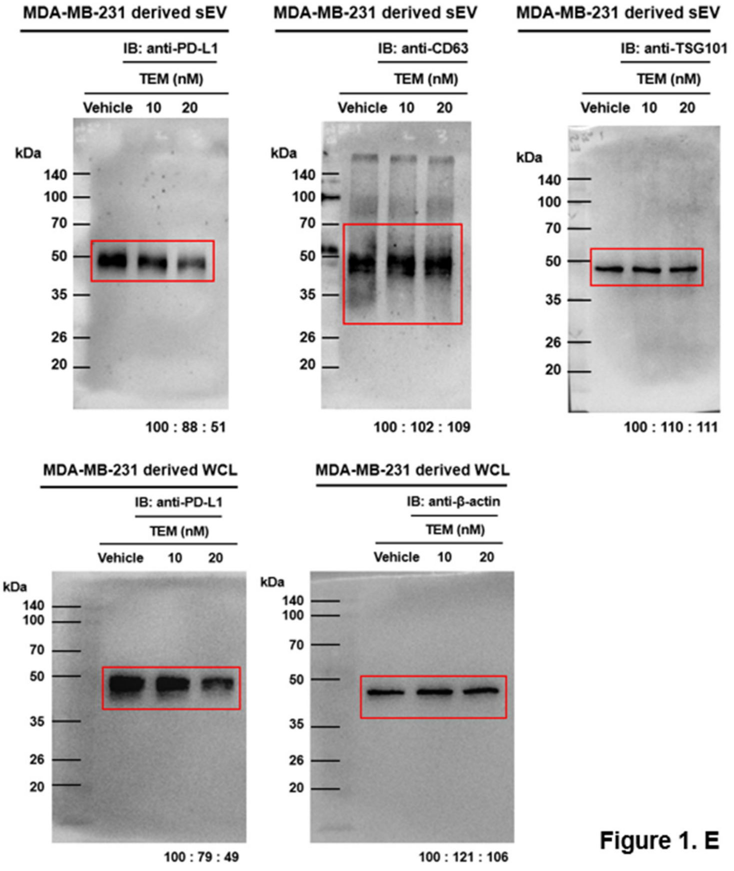


**Figure 1. E**

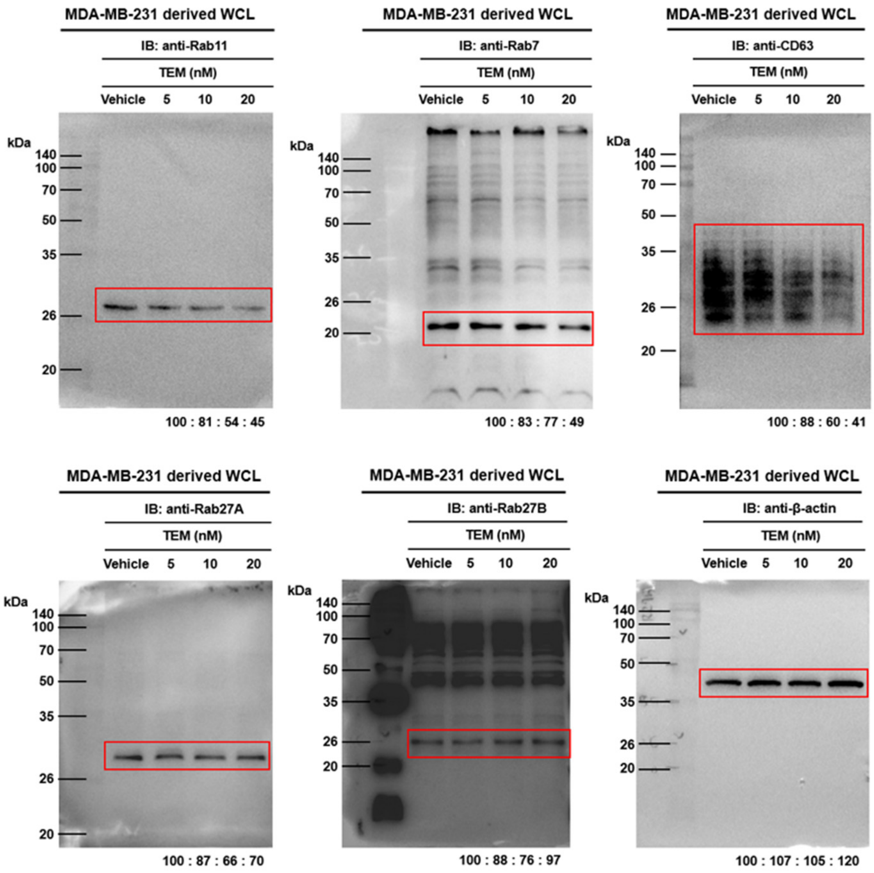


**Figure 2. A**

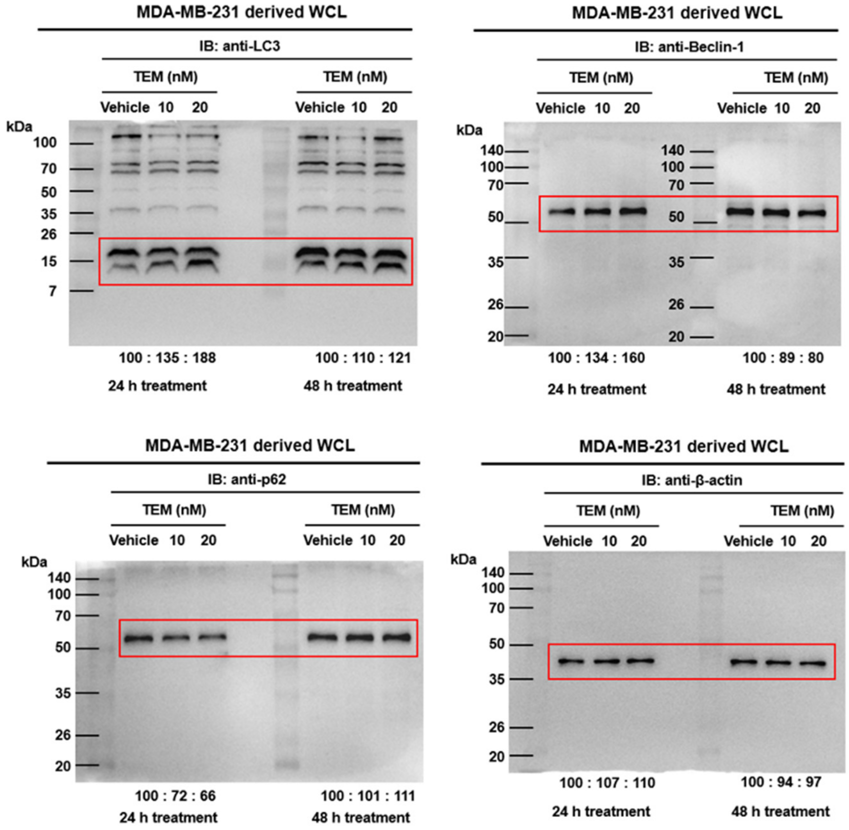


**Figure 2. B**

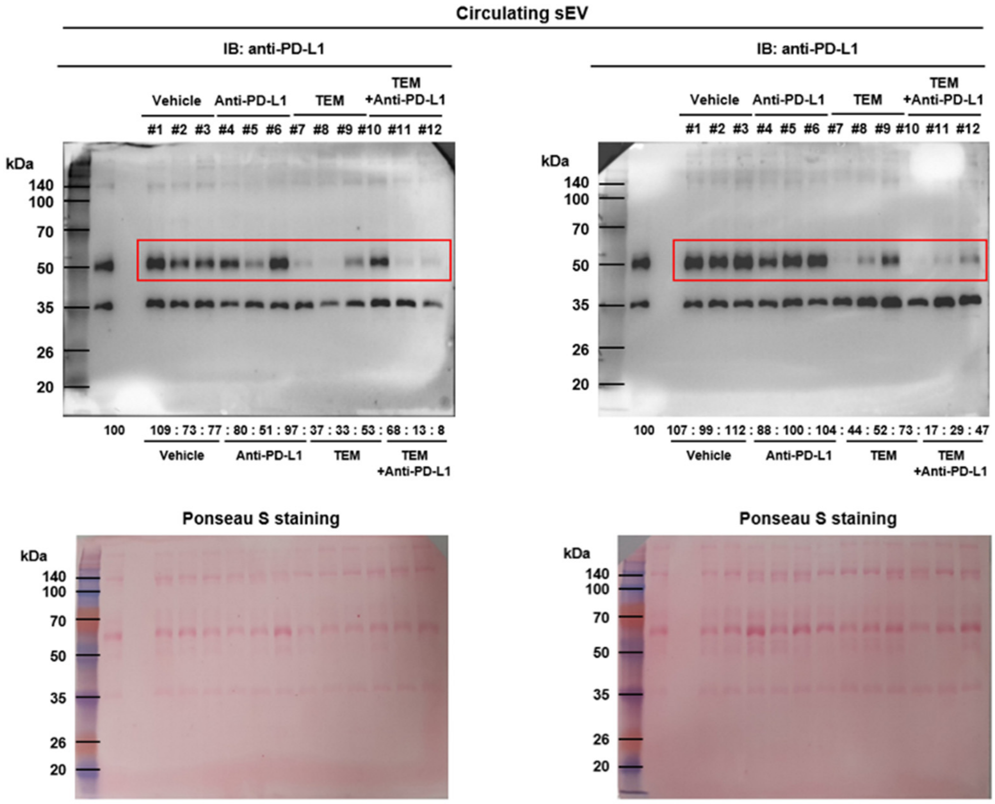


Figure 4. E

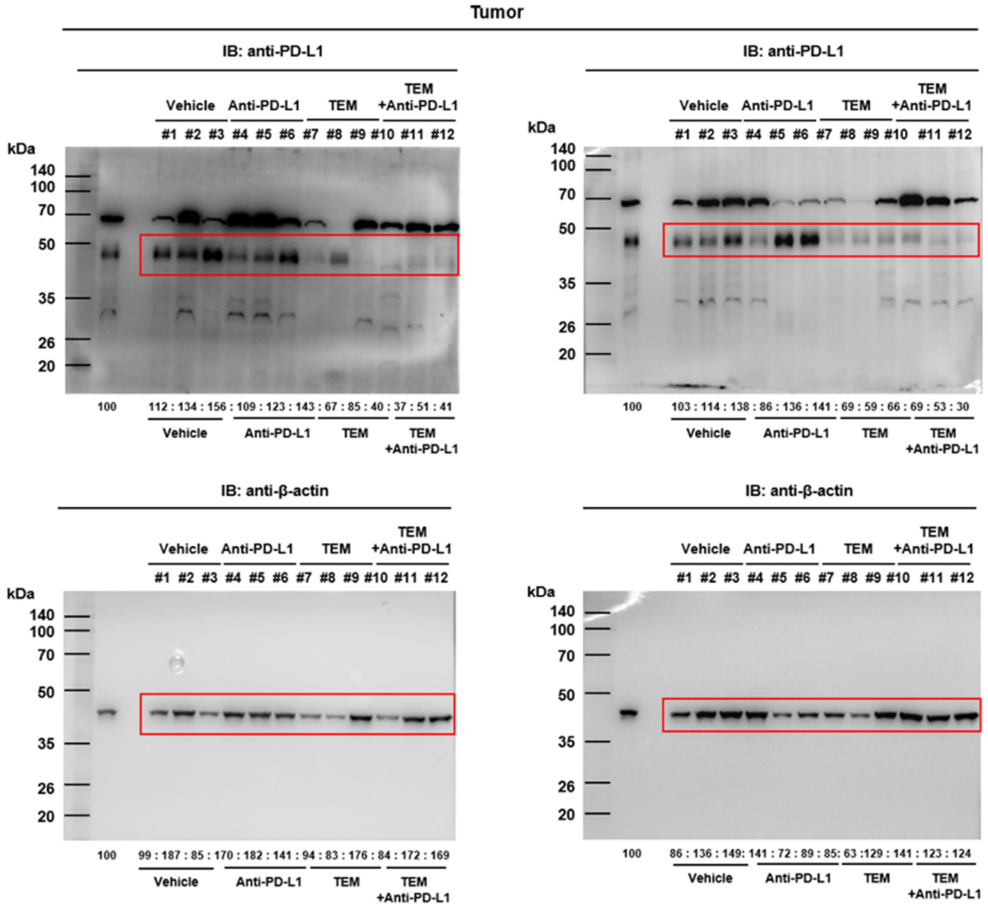


Figure 4. F

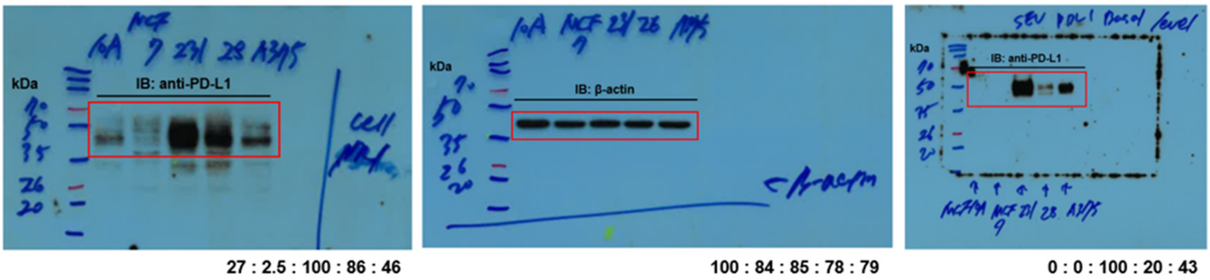


**Figure S2. A**

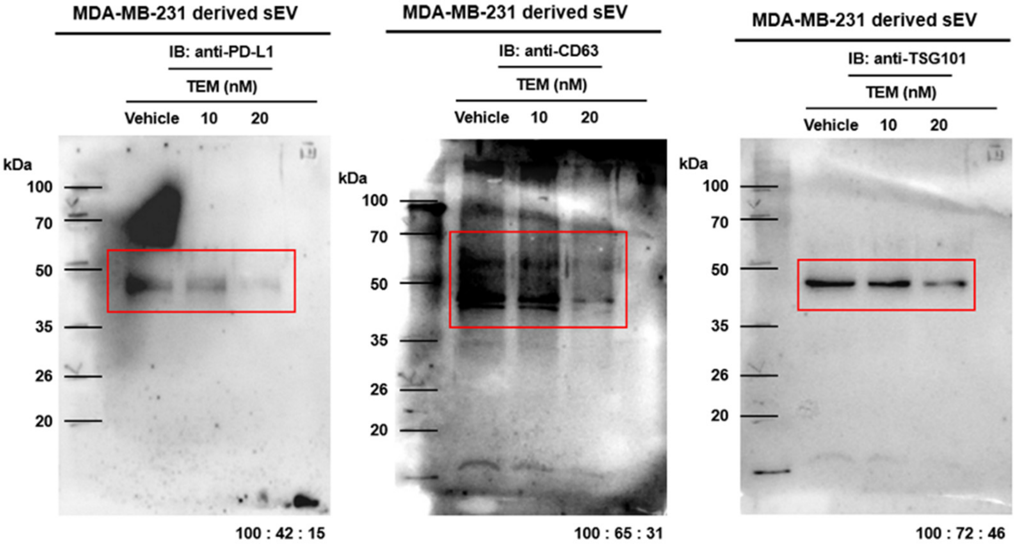


**Figure S2. C**

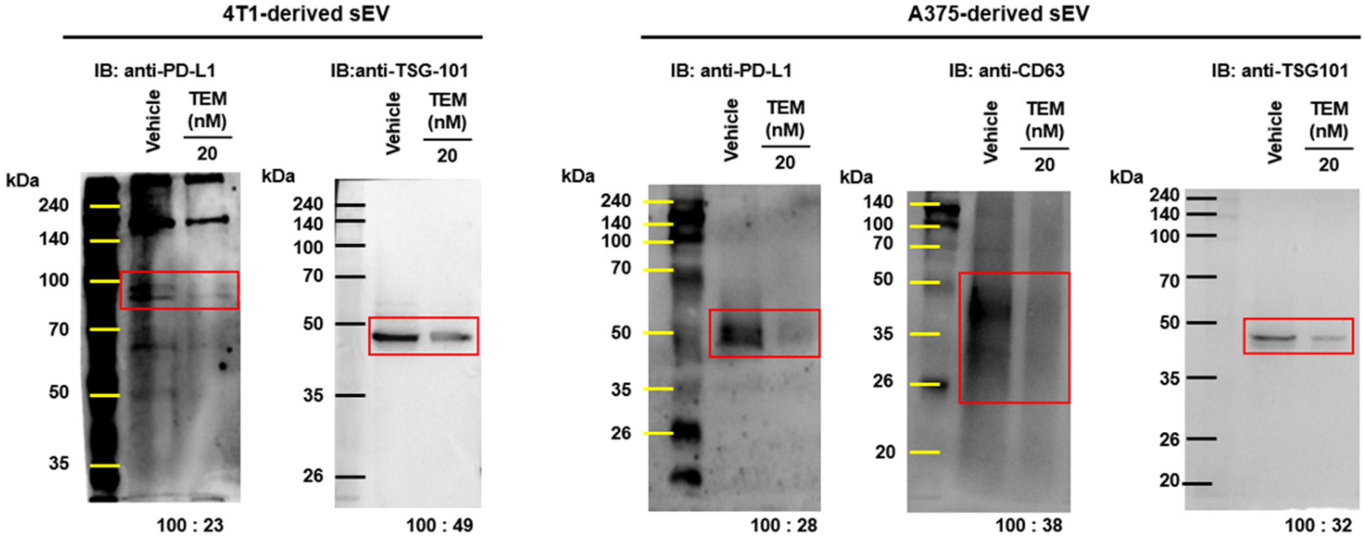


**Figure S3. A**

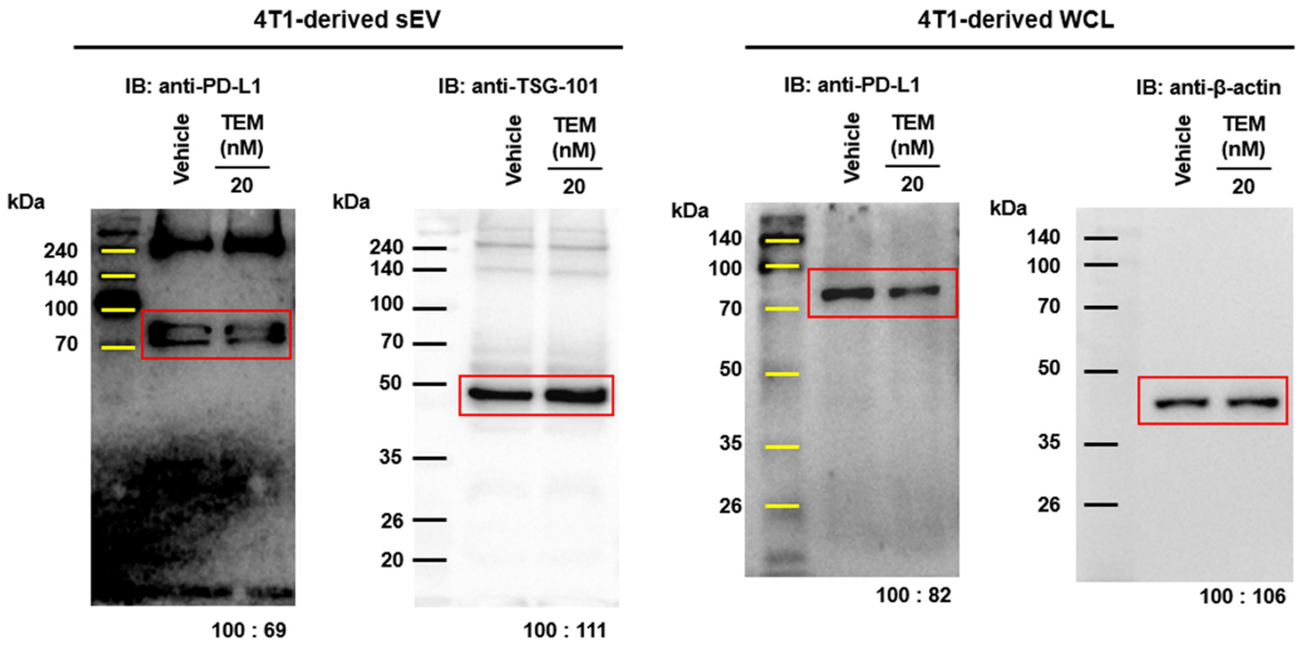


Figure S3. C

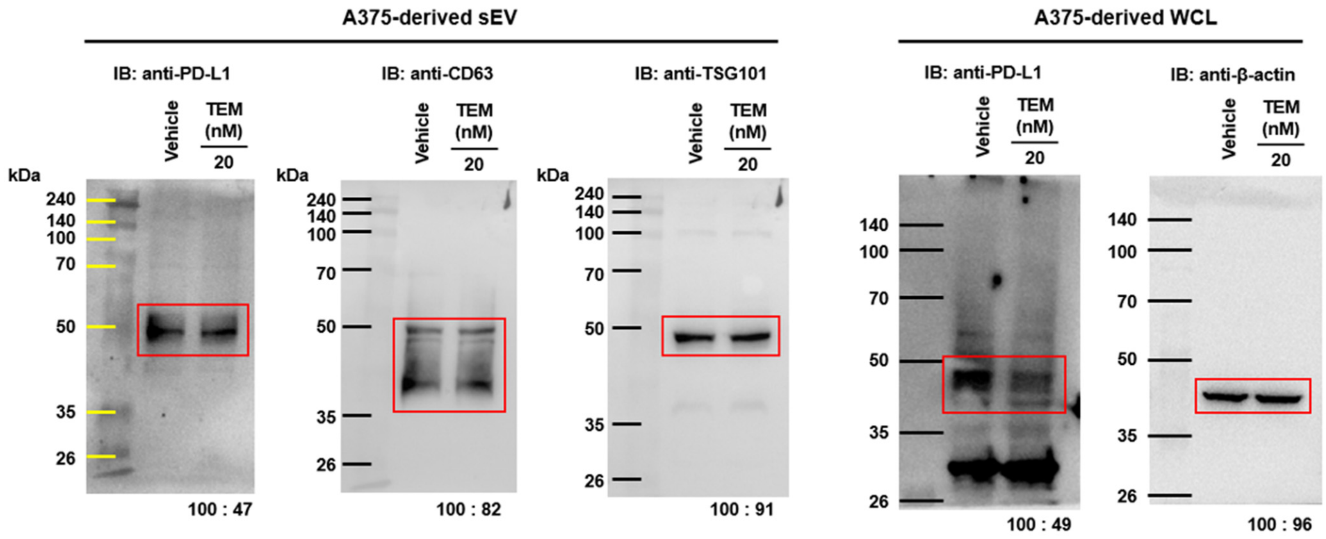


Figure S3. C

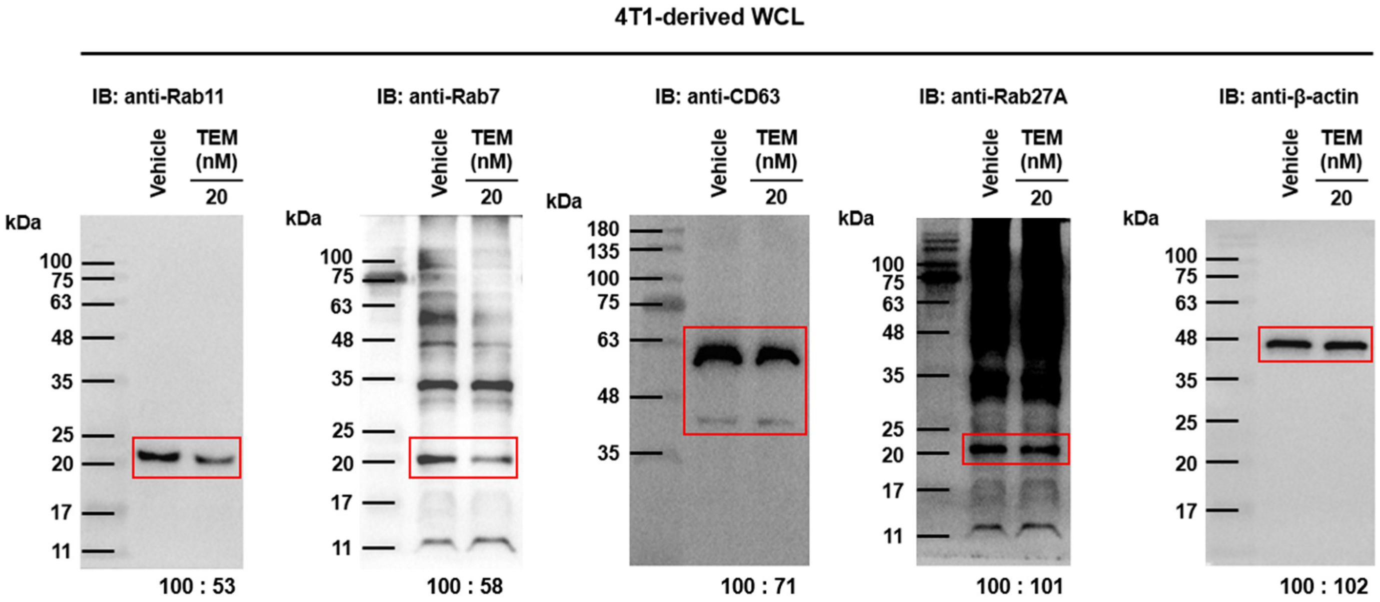


Figure S4. A

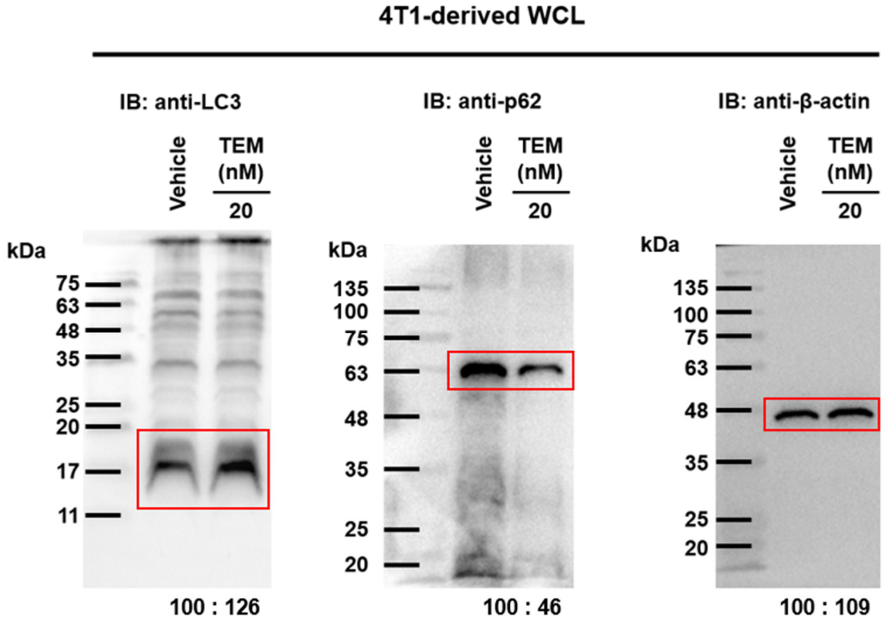


Figure S4. B

**Figure S1.** Original blots of western blot analyses in this paper. The original blots of western blots were detected by immunoblotting and indicated in the corresponding protein regions by the red boxes.

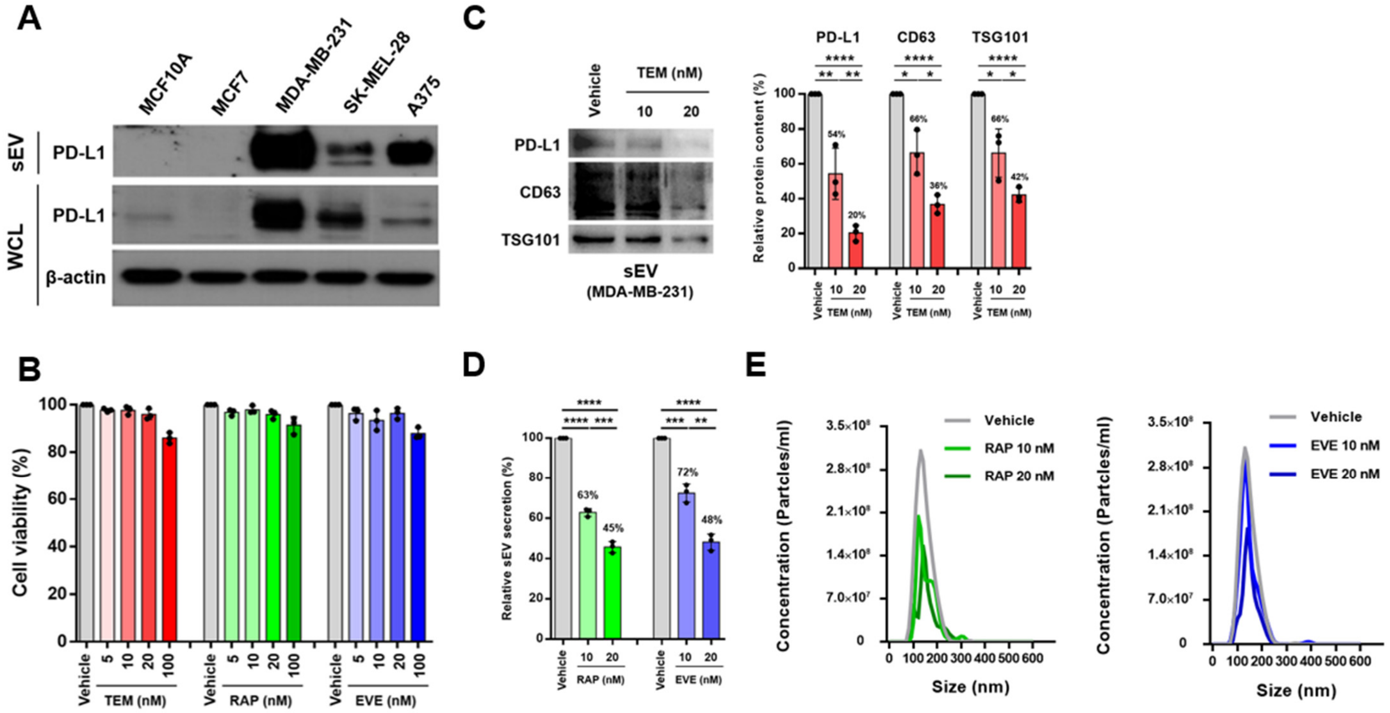


**Figure S2.** mTOR inhibitors inhibit the secretion of sEV from MDA-MB-231 cells. (A) Immunoblots of sEV PD-L1 (top) and cellular PD-L1 (bottom) in three representative human breast cell lines [MCF10A (normal), MCF7, and MDA-MB-231 (cancer)] and two melanoma cell lines (SK-MEL-28 and A375). sEV proteins from approximately 2.4 × $10^7$ cells were loaded per lane. (B) The viability of MDA-MB-231 cells by treatment of the variously indicated mTOR inhibitors concentrations for 48 h ($n = 3$). (C) Immunoblots of various proteins in sEVs from MDA-MB-231 cells with or without treatment with various concentrations of TEM ($n = 3$). sEV proteins from approximately 1.2 × $10^7$ cells were loaded per lane. (D) The relative percentage of secreted sEV from MDA-MB-231 cells with or without treatment of RAP or EVE by nanoparticle tracking analysis (NTA) ($n = 3$). (E) A representative graph of the size distribution of secreted sEV from MDA-MB-231 cells with or without treatment of RAP or EVE. The data are presented as means ± standard deviation (SD) using an unpaired two-tailed student's t-test. *$p < 0.05$, **$p < 0.01$, ***$p < 0.001$, and ****$p < 0.0001$, respectively; ns, not significant.

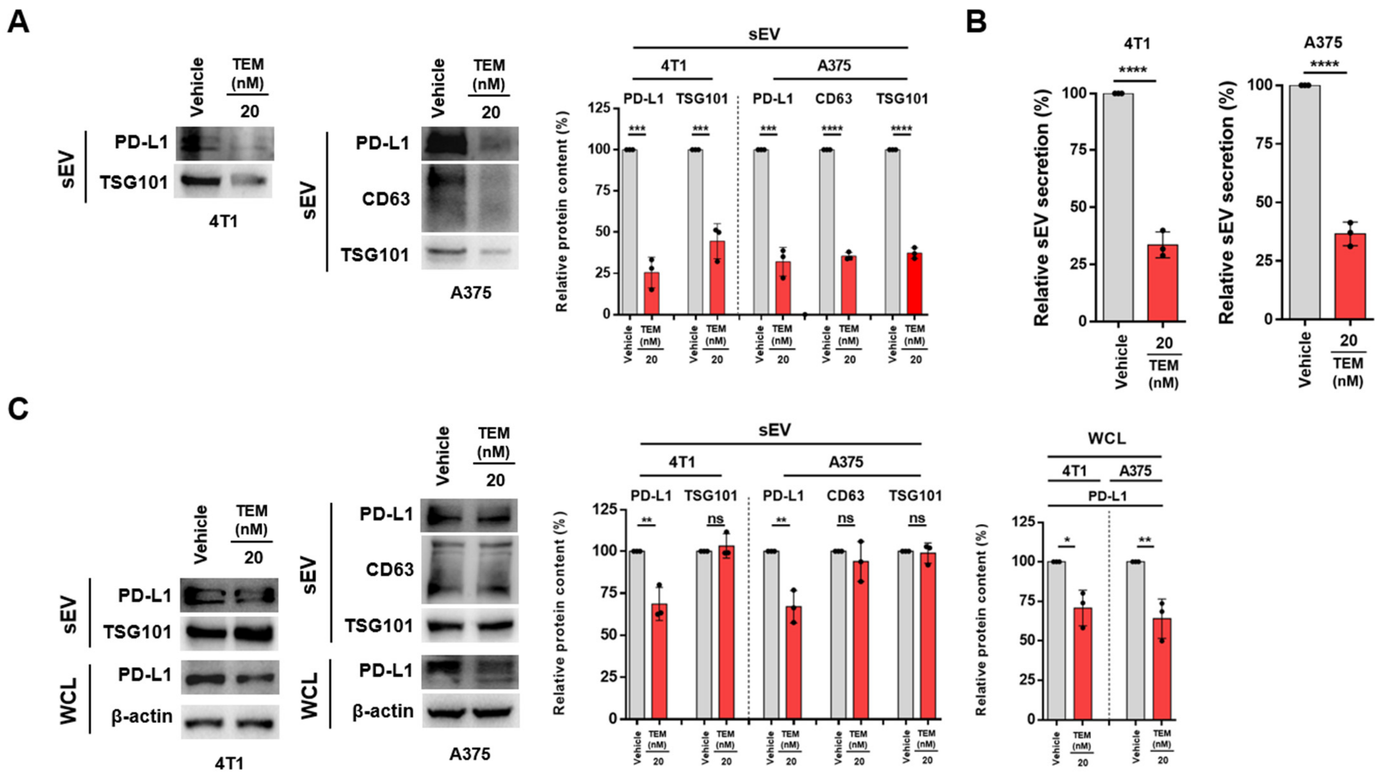


**Figure S3.** TEM reduces sEV PD-L1 secretion via the additive effect of inhibiting sEV secretion and cellular PD-L1 expression in the various cancer cell lines. (A) Immunoblots of various proteins in sEVs from 4T1 murine breast cancer cells and A375 human melanoma cells with or without treatment of TEM ($n$ = 3). sEV proteins from approximately $1.2 \times 10^7$ cells were loaded per lane. (B) The relative percentage of secreted sEV from 4T1 murine breast cancer cells and A375 human melanoma cells with or without treatment of TEM by nanoparticle tracking analysis (NTA) ($n$ = 3). (C) Immunoblots of various proteins in sEV (top) and whole cell lysate (WCL) (bottom) from 4T1 murine breast cancer cells and A375 human melanoma cells with or without treatment of TEM ($n$ = 3). An equal protein weight (3 μg) of the sEV was loaded per lane. β-actin was used as the loading control. The data are presented as means ± standard deviation (SD) using an unpaired two-tailed student's t-test. *$p$ < 0.05, **$p$ < 0.01, ***$p$ < 0.001, and ****$p$ < 0.0001, respectively; ns, not significant.

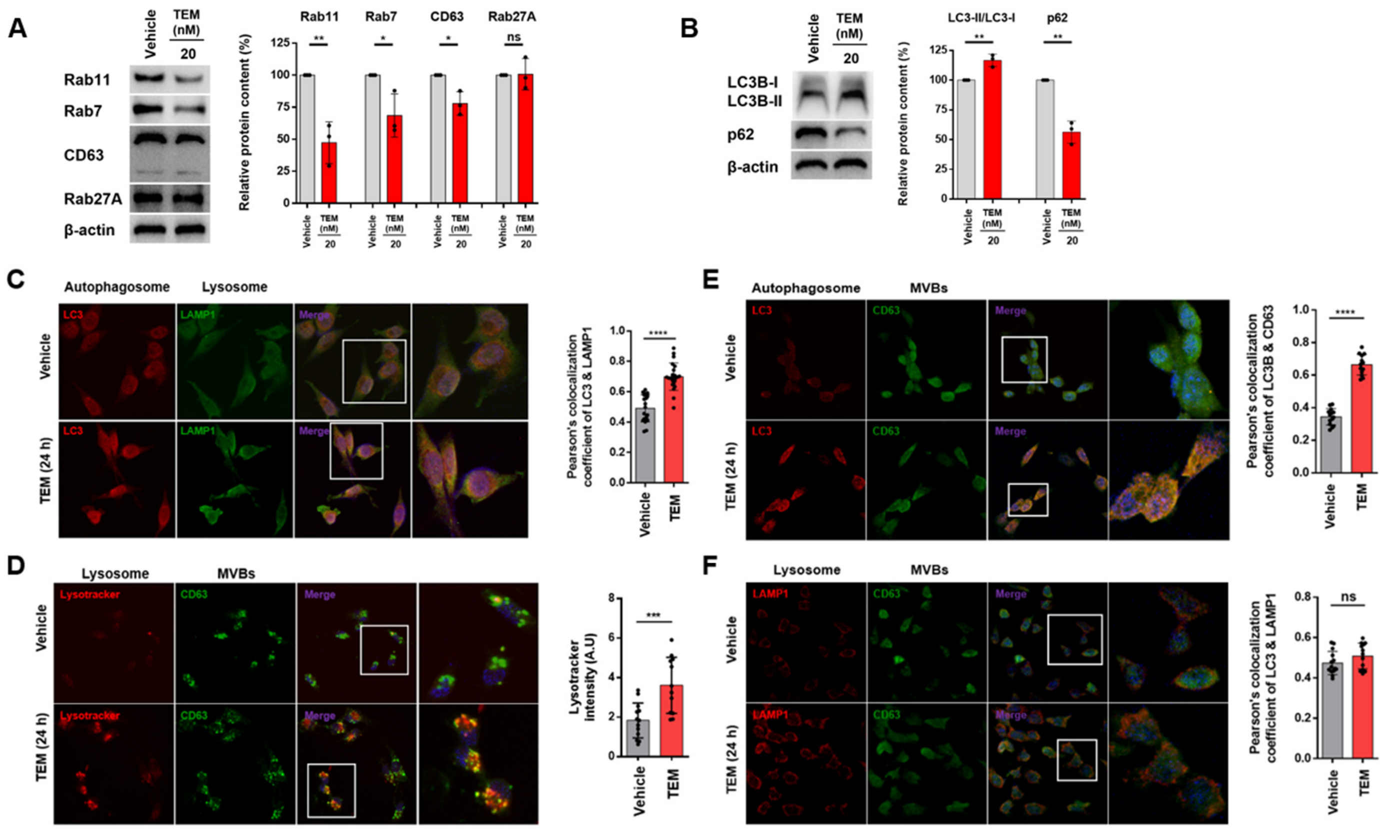


**Figure S4.** TEM induces activation of autophagy. (A-B) The immunoblots of various proteins are associated with (A) small extracellular vesicle (sEV) secretion and (B) autophagy in 4T1 cells. (left) ($n$ = 3). β-actin was used as the loading control. The quantitative analysis of the relative protein expression (right). (C) Confocal microscopy image. Colocalization of LC3 (autophagosome) and LAMP1 (lysosome) in MDA-MB-231 cells by TEM treatment. (D) Confocal microscopy image of lysosome activity in MDA-MB-231 cells by TEM treatment. (E) Confocal microscopy image. Colocalization of LC3 (autophagosome) and CD63 [(multivesicular bodies (MVBs)] in 4T1 cells by TEM treatment. (F) Confocal microscopy image. Colocalization of LAMP1 (Lysosome) and CD63 (MVBs) in 4T1 cells by TEM treatment. The colocalization was estimated using Pearson's correlation coefficient. The data are presented as means ± standard deviation (SD) using an unpaired two-tailed student's t-test. *$p < 0.05$, **$p < 0.01$, ***$p < 0.001$, and ****$p < 0.0001$, respectively; ns, not significant.

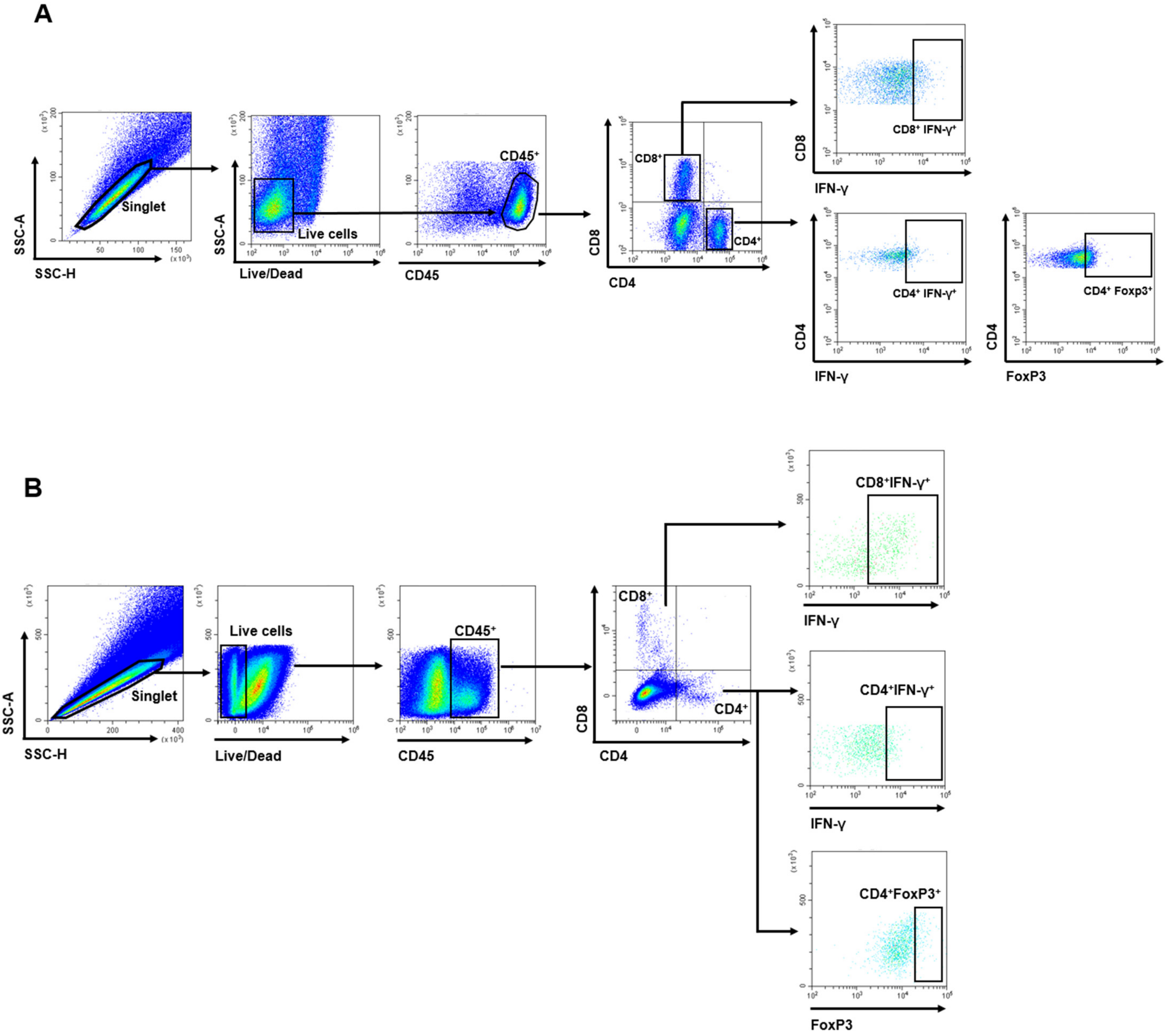


**Figure S5.** The gating strategy used in flow cytometry analysis. The gating strategy for immunophenotyping the cells in (A) DLN and (B) tumor. A time gate was initially applied to exclude any doublets, and dead cells, then all cells were categorized based on indicated antibodies.
